# The Incidence of Perioperative Hypotension in Patients Undergoing Major Abdominal Surgery with the Use of Arterial Waveform Analysis and the Hypotension Prediction Index Hemodynamic Monitoring—A Retrospective Analysis

**DOI:** 10.3390/jpm14020174

**Published:** 2024-02-02

**Authors:** Jakub Szrama, Agata Gradys, Tomasz Bartkowiak, Amadeusz Woźniak, Zuzanna Nowak, Krzysztof Zwoliński, Ashish Lohani, Natalia Jawień, Piotr Smuszkiewicz, Krzysztof Kusza

**Affiliations:** Department of Anesthesiology, Intensive Therapy and Pain Management, Poznan University of Medical Sciences, 60-355 Poznan, Polandashish.lohani@usk.poznan.pl (A.L.); piotr.smuszkiewicz@usk.poznan.pl (P.S.); krzysztof.kusza@usk.poznan.pl (K.K.)

**Keywords:** perioperative hypotension, hemodynamic monitoring, hypotension prediction index, arterial pressure-based cardiac output

## Abstract

Intraoperative hypotension (IH) is common in patients receiving general anesthesia and can lead to serious complications such as kidney failure, myocardial injury and increased mortality. The Hypotension Prediction Index (HPI) algorithm is a machine learning system that analyzes the arterial pressure waveform and alerts the clinician of an impending hypotension event. The purpose of the study was to compare the frequency of perioperative hypotension in patients undergoing major abdominal surgery with different types of hemodynamic monitoring. The study included 61 patients who were monitored with the arterial pressure-based cardiac output (APCO) technology (FloTrac group) and 62 patients with the Hypotension Prediction Index algorithm (HPI group). Our primary outcome was the time-weighted average (TWA) of hypotension below < 65 mmHg. The median TWA of hypotension in the FloTrac group was 0.31 mmHg versus 0.09 mmHg in the HPI group (*p* = 0.000009). In the FloTrac group, the average time of hypotension was 27.9 min vs. 8.1 min in the HPI group (*p* = 0.000023). By applying the HPI algorithm in addition to an arterial waveform analysis alone, we were able to significantly decrease the frequency and duration of perioperative hypotension events in patients who underwent major abdominal surgery.

## 1. Introduction

The fundamental aspect of anesthetics perioperative management is to maintain hemodynamic stability, with special attention on the avoidance or reduction of episodes of intraoperative hypotension (IH) [1]. A lot of research was performed on the negative outcomes that can result from low blood pressure during surgery, but there is no clear agreement on what constitutes as hypotension [2]. This makes it difficult to compare studies and establish guidelines for optimal blood pressure management in different surgical settings.

In addition, recent research supports the concept of individualized patient blood pressure management rather than using a universal one-size-fits-all rule, with the personalized approach enabling a decrease in perioperative organ dysfunction [3].

The incidence of intraoperative hypotension is related to an increased rate of perioperative morbidity and mortality [4,5,6,7,8,9,10,11,12,13,14,15,16,17]. Even a short period of hypotension can be related to an increased risk of postoperative stroke [14], myocardial injury [7,8,15,16] and acute kidney injury [7,8,9]. The incidence of postoperative organ injury can be affected by the severity and duration of the hypotensive event [7,8]. A meta-analysis of a group of more than 120,000 patients showed an association between the incidence of hypotension and the increased 30-day postoperative mortality and the rate of major cardiac complications [17]. Another study performed in a group of 33,000 patients showed a relationship between episodes of severe hypotension with MAP < 55 mmHg with a duration of 1–5 min and the risk of myocardial and kidney injury [7].

The use of advanced hemodynamic monitoring and the application of perioperative goal-directed therapy seemed to be an appropriate management aimed at reducing the incidence of IH. However, recent meta-analyses evaluating the effects of perioperative goal-directed therapy and advanced hemodynamic monitoring did not show any beneficial effects on the reduction of postoperative complications and mortality [18,19,20].

Despite the use of advanced hemodynamic monitoring and goal-directed therapy protocols, our management of perioperative hypotension is a reactive approach. We intervene when the hypotension already occurs and the best we can do is to shorten the duration of the hypotensive event. A recently introduced algorithm named the Hypotension Prediction Index (HPI; Edwards Lifesciences, Irvine, CA, USA) might change the management of IH into a more proactive approach. The HPI technology is a machine learning algorithm analyzing the arterial pressure waveform and giving the clinician a unitless number, ranging from 0 to 100, and informing the anesthesiologist about the likelihood that within 5–15 min, a hypotensive event with a MAP < 65 mmHg lasting for more than 1 min will occur despite the patients still being hemodynamically stable [21]. The higher the value of the HPI, the greater the risk of hypotensive events. The sensitivity and specificity of predicting hypotension with this model are 92% and 92% at 5 min, 89% and 90% at 10 min and 88% and 87% at 15 min, respectively, before the occurrence of hypotension [22]. However, these values might be overestimated as a result of the bias regarding data selection, which could affect the validity and reliability of the HPI algorithm [23,24]. In some studies, the HPI technology seemed to be less reliable. The AUC value for predicting hypotension in patients undergoing living donor liver transplantation at five minutes was 0.810, and for predicting hypotension at 10 and 15 min, 0.726 and 0.689, respectively [25].

There is an increasing number of clinical studies showing a reduced rate of perioperative hypotension with the use of HPI technology [26,27,28,29,30]. However, recently, some issues regarding the true usefulness of the HPI technology and its very close relationship with MAP were raised [31,32].

Most of those studies implementing the HPI technology compared it with the standard invasive blood pressure monitoring, showing, in most cases, a substantial reduction in the episodes of hypotension. However, one of the remaining questions is whether the HPI technology will do better than advanced hemodynamic monitoring applying arterial pressure-based cardiac output (APCO) evaluation. We are also still lacking studies which could prove the clinical benefits of reducing perioperative morbidity with the HPI technology. The aim of this retrospective study was the comparison of the amount of perioperative hypotension in patients undergoing major abdominal surgery having either APCO hemodynamic monitoring with the FloTrac sensor or the HPI hemodynamic monitoring.

## 2. Materials and Methods

This single-center retrospective study was performed in the Department of Anesthesiology, Intensive Care and Pain Management of the Poznan University of Medical Science Hospital between September 2022 and February 2023. The Bioethics Committee of the Poznan University of Medical Sciences approved the study protocol (number 559/220). Due to retrospective nature of the study, informed consent was waived from the study subjects.

The study analysis included all moderate and high-risk patients undergoing major abdominal surgery during the study period with the perioperative application of advanced hemodynamic monitoring. The inclusion criteria were age above 18 years, elective major abdominal surgery (e.g., intestinal surgery, gastrectomy, pancreatoduodenectomy), the measurement of the invasive blood pressure with the insertion of an arterial line, the use of the advanced hemodynamic monitoring, the duration of the surgery > 120 min. The exclusion criteria were age < 18 years, emergency surgery, duration of surgery < 120 min, patients with arrhythmia impairing arterial pressure, cardiac output monitoring, heart failure with ejection fraction < 35%, severe aortic and mitral stenosis.

**Figure 1:**
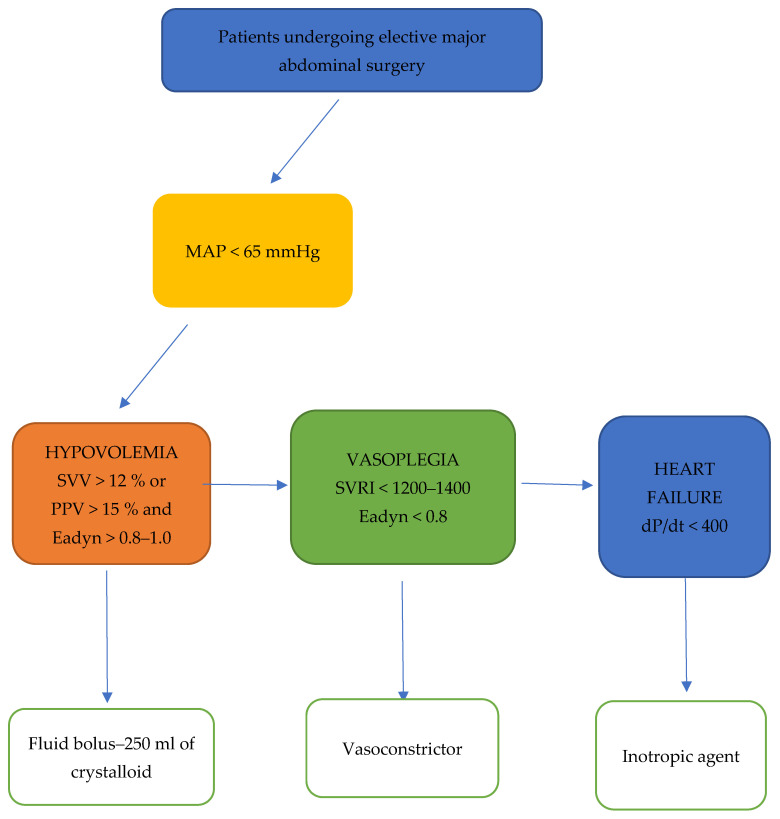
GDT protocol applied in the FloTrac group.

During the study period, the decision to use advanced hemodynamic monitoring during anesthesia was based on our local institution’s guidelines based on the type of surgery and the risk assessment during patient premedication. The type of hemodynamic monitoring, FloTrac sensor vs. HPI sensor (Acumen IQ), was made by the attending anesthesiologist. After induction of anesthesia, an arterial line was placed in the radial artery, and the advanced hemodynamic monitoring was started immediately. In the FloTrac group, the attending anesthesiologist applied crystalloids, vasopressors and inotropes according to our institution’s GDT protocol, presented in Figure 1.

**Figure 2:**
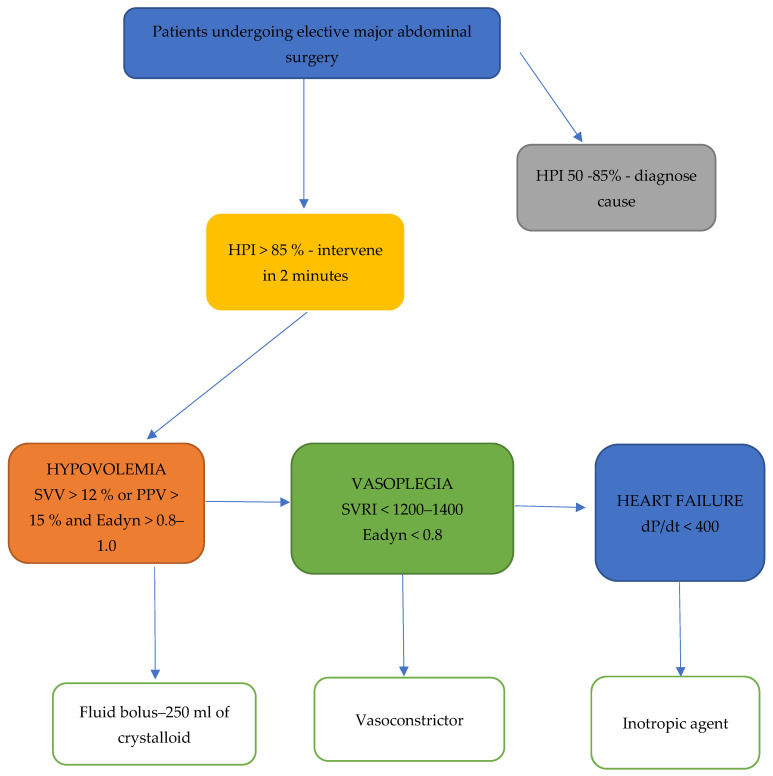
Hemodynamic protocol applied to patients with the HPI hemodynamic monitoring.

The HPI algorithm gives a unitless number, ranging from 0 to 100, which informs the clinician about the likelihood of a hypotensive event. The lower the HPI value, the lower the risk of the hypotensive event [21]. When the HPI value exceeds 85, an alarm appears on the screen informing about the upcoming hypotensive event. The clinician should then follow the information from the secondary screen of the Hemosphere platform that provides hemodynamic data on the preload, contractility and afterload in order to avoid future hypotensive events [21]. In the HPI group, the treatment of hemodynamic instability was based on our institution protocol implementing hemodynamic parameters derived from the Hemosphere secondary screen (Figure 2). In both groups, infusion of colloids and blood products was the decision of the attending anesthesiologist.

The primary endpoint of the study was the time-weighted average of hypotension below 65 mmHg (TWA − MAP < 65 mmHg). The time-weighted average of hypotension below 65 mmHg is the combination of the severity and the duration in relation to the time of surgery [25]. It is calculated as follows: TWA − MAP < 65 mmHg—time weighed average = (depth of hypotension in millimeters of mercury below a MAP of 65 mmHg × time in minutes spent below MAP of 65 mmHg)/total duration of operation in minutes). The hypotensive event is defined as MAP below 65 mmHg for more than one minute. Secondary endpoints consisted of average number of hypotensive events for MAP < 65 mmHg and < 50 mmHg, cumulative and average duration of hypotensive events for MAP < 65 mmHg and < 50 mmHg, the number of patients with hypotensive events < 65 mmHg and < 50 mmHg and TWA for MAP < 50 mmHg.

### Statistical Analysis

The perioperatively collected hemodynamic data were downloaded retrospectively from the HemoSphere platform and analyzed using the Acumen Analytics Software Version 1.0.22 (Edwards Lifesciences Corp., Edwards Lifesciences, Irvine, CA, USA). Categorical data are presented as frequencies with percentages and were analyzed with Fisher’s exact test. The quantitative variables were tested for normal distribution with the Shapiro–Wilk test. Data with normal distribution are presented as means ± standard deviation (SD); otherwise, data are presented as median and 25th (Q1) and 75th (Q3) percentiles. Linear variables were analyzed using the Student’s unpaired *t*-test in normal distributed variables and the Mann–Whitney U-test in nonnormally distributed variables. *p*-values less than 0.05 were considered statistically significant. Statistical analysis was performed with MedCalc^®^ Statistical Software version 20.115 (MedCalc Software Ltd., Ostend, Belgium; 2022).

## 3. Results

One hundred twenty-three patients undergoing major abdominal surgery were included in the analysis between September 2022 and February 2023. Sixty-one patients constituted the FloTrac group and sixty-two the HPI group. The analyzed groups did not differ in the basic demographic data, including the preoperative ASA status, comorbidities, administered drugs and type of surgery (Table 1). Similarly, there were no differences in the duration of surgery, perioperative fluid status or noradrenaline infusion (Table 2).

The primary endpoint of the study, the median TWA of MAP < 65 mmHg for the FloTrac group, was 0.31 mmHg in comparison to 0.09 mmHg in the HPI group (*p* = 0.000009) (Table 3). More patients in the FloTrac group experienced episodes of hypotension below 65 mmHg than in the HPI group; however, it was not statistically significant (Table 3). The median value of the number and the duration of the hypotensive events were significantly higher in the FloTrac group. Patients in the HPI group spent less time in hypotension in comparison to the FloTrac group, with a median value of 4 min in comparison to 12 min in the FloTrac group (*p* = 0.000078). The percentage of total monitoring time in hypotension was also smaller in the HPI group in comparison to the FloTrac patients (2.1% vs. 6.4%, *p* = 0.00023). The data on hypotension with MAP < 65 mmHg are summarized in Table 3.

A similar analysis was performed for the episodes of severe hypotension with MAP < 50 mmHg. The overall incidence of severe hypotension was very low. The mean value for TWA for MAP < 50 mmHg in the FloTrac group was 0.12 ± 0.31, and for the HPI group, 0 ± 0.01 mmHg (*p* = 0.0018). Patients in the FloTrac group had a higher average number and longer duration of severe hypotensive events in comparison to the HPI group. Total time in severe hypotension in relation to the total monitoring time was also longer for the FloTrac group in comparison to the HPI group. The data on the severe hypotension are summarized in Table 4.

## 4. Discussion

The results of this retrospective study confirm the clinical usefulness of the HPI algorithm in the reduction of hypotensive events in the perioperative period. The TWA, which includes both the duration and severity of the hypotension events, was significantly lower in the HPI group in comparison to the FloTrac group (0.09 vs. 0.31 mmHg). Similarly, the rate of severe hypotension with MAP < 50 mmHg was almost eliminated with the use of HPI monitoring. A study performed on 60 patients undergoing major oncological gynecological procedures showed a statistically non-significant reduction in the rate of severe hypotension with the use of HPI technology in comparison to the control group with APCO monitoring [33]. To our knowledge, this is the biggest study analyzing the usefulness of the HPI technology in patients undergoing major abdominal surgery and the only study reporting a significant reduction of the episodes of severe hypotension with MAP < 50 mmHg with the use of the HPI technology.

Since the introduction and validation of the HPI technology in 2018 [22], there have been few studies published analyzing the usefulness of the HPI algorithm in different patient populations [26,27,28,29,30]. One of the first randomized clinical trials evaluating the effect of the HPI technology on the rate of hypotension measured by the TWA was a study by Wijnberge, which compared standard care with the use of the HPI algorithm in 68 patients undergoing mainly gastrointestinal surgery [26]. The TWA for the HPI group was 0.10 mmHg in comparison to 0.44 mmHg in the control group. These results seem to be comparable with our retrospective analysis of the 123 patients, with the median TWA for the HPI group being 0.09 mmHg and for the Flotrac group 0.31 mmHg. A slightly lower value of TWA in the FloTrac group in our study in comparison to the “control” group of the HYPE study might show the benefits of reducing hypotension with the use of GDT protocol and the APCO technology.

Similar results to our study were published in a retrospective analysis by Grundman from 2021 [34], where the authors compared 100 adult consecutive patients undergoing moderate or high-risk noncardiac surgery with invasive blood pressure monitoring. A total of 50 of those patients received hemodynamic monitoring with the FloTrac technology, and 50 patients were monitored with the HPI technology. The majority of the patients in that study were classified as ASA III status, which is similar to the population from our study. However, only 28 patients in the mentioned study had gastrointestinal surgery performed, while in our cohort of patients, all 123 patients had major abdominal surgery.

The authors of the study performed by Grundman et al. were able to show a significant reduction in TWA of hypotension below 65 mmHg in the HPI group. Similarly to our results, there was also a significant reduction in the median duration of the hypotensive events (1 vs. 2.75 min, in the HPI and FloTrac groups, respectively) and the cumulative duration of the hypotension below 65 mmHg per patient (1 vs. 10.3 min and 1.14% vs. 7.44% of the monitored time, in the HPI and Flotrac group, respectively). In our study, the use of the HPI technology also allowed a reduction in the duration of each hypotensive event and the median cumulative duration of the hypotension during the monitored time. The authors of the mentioned study report a very low number of incidence of severe hypotension below < 50 mmHg. In our study, we showed a significant reduction in the TWA of MAP < 50 mmHg in the HPI group in comparison to the FloTrac group. The use of the HPI technology in comparison to the FlowTrac allowed a 90% reduction in the duration of hypotension < 50 mmHg. The mean value of the cumulative time spent with severe hypotension in the HPI group was 0.3 min, which constituted 0.1% of total monitoring time.

Another retrospective study comparing the HPI technology with the FloTrac monitoring is the study published in 2022 by Solares [35]. However, the patient population seemed to be “healthier”, as just one-third of patients were classified as ASA III. Again, most of those patients had orthopedic surgery, with gastrointestinal surgery performed only in 20 patients. Similarly to our results, the authors were able to show a reduction in the TWA of hypotension < 65 mmHg with median values of 0.09 in the HPI group and 0.23 mmHg in the FloTrac group, respectively. Similarly to the previously mentioned study by Grundman [34] and the results of our study, Solares and colleges proved a significant reduction in the duration of the hypotensive events in relation to the total surgery/monitoring time, which constituted 5.1% of total procedure time in the Flotrac group and 2.4% of total procedure time in the HPI cohort. In our study, the values were comparable and also favorable for the HPI group patients, with 2.1% of total monitoring time in the HPI group and 6.4% in the FloTrac group, respectively. In contrast to our study, Solares et al. do not give information on the rate of hypotension < 50 mmHg with the use of both these advanced hemodynamic monitoring technologies.

However, it is not possible to discuss the issue of the Hypotension Prediction Index without its possible limitations and concerns present in the literature regarding its true usefulness [36]. A pilot randomized trial conducted by Maheshwari et al. proved no benefits in the reduction of hypotension in 214 surgical patients with the use of HPI technology in comparison to standard care. Among the conclusions, the authors pointed out that the possible reason for no hypotension reduction was the fact that the anesthesiologists did not follow the HPI alerts due to short warning time, complex treatment algorithms, or simply ignoring the alert [28]. A prospective observational study performed on a group of 20 adult patients undergoing living donor liver transplantation showed that the HPI technology applied in this study predicted hypotension with moderate-to-low accuracy. To analyze the HPI technology performance, the authors calculated the area under the curve (AUC) of the receiver operating characteristic curve. According to the results of the study, the performance of the HPI in the living donor liver transplantation was inferior to the previously reported values in patients undergoing major surgery. Previous studies in noncardiac surgeries showed very good performance of the HPI technology with AUC values greater than 0.95 at 5, 10 and 15 min before the occurrence of hypotension [22]. The AUC value for predicting hypotension with the HPI in patients undergoing living donor liver transplantation at five minutes was 0.810, and for predicting hypotension at 10 and 15 min, 0.726 and 0.689, respectively [25]. Interestingly, the MAP AUC for the ROC curves for predicting a hypotensive event in the subsequent five minutes was 0.816, the subsequent 10 min was 0.728 and the subsequent 15 min was 0.691 [25]. The ROC curves for HPI and MAP overlap completely, indicating that there is no difference in specificity and sensitivity at any point. This is consistent with the high correlation between the two variables, which perform identically in predicting hypotension [23]. The lower values of the AUC for predicting hypotension with the HPI technology in the living donor liver transplant patients can be explained by factors specific to liver transplantation, which is considered a surgical procedure with a high level of hemodynamic instability in comparison with other noncardiac surgeries [25]. However, regarding the recent concerns regarding the HPI’s predictive abilities, the lower values of the AUC might reveal its real-life performance [23]. A major concern was raised regarding the bias of data selection while introducing and developing the HPI algorithm [23,24]. The analysis of the methodology of the index publication by Hatib [21] revealed that if hypotension was the outcome, MAP could be any value, but if the outcome was non-hypotension, MAP was always above 75 mmHg [23,24]. This means that the HPI model is trained on data that have a label leakage problem. If the current MAP is below 75 mmHg, the model will always predict hypotension in the future because it has learned this from the data. A model based on such data will not capture the true relationship between hypotension and other features, such as waveform patterns that indicate changes in vascular tone. The mentioned bias of data selection was also present in most of the subsequent HPI validation studies [23].

Interestingly, the authors of a study on the HPI performance in comparison to other standard hemodynamic parameters showing high accuracy of the HPI algorithm in predicting hypotension [37] published an erratum almost 3 years after the original publication in which they report an error in the main conclusion due to differences in the data analyses of HPI and MAP [37]. To compare HPI and MAP, the authors used different criteria for selecting non-hypotensive events. For HPI, the authors included only events where the ABP waveform before the hypotension had MAP > 75 mmHg. For MAP, events where the ABP waveform before the hypotension had either MAP > 75 mmHg or MAP < 75 mmHg were included. Therefore, the ROC curves and AUCs for HPI and MAP are not directly comparable. This contradicts the main conclusion of the original paper, which claimed that HPI was better than MAP in predicting episodes of MAP < 65 mmHg [37]. The data selection bias during the development of the HPI technology made this algorithm learn almost solely from MAP and not from the arterial waveform patterns [24].

There is a correlation between MAP and HPI, described as the mirror effect [31]. When the HPI value reaches 85, which is the default alarm level for the intervention, the corresponding MAP is approximately 72 to 76 mmHg, so using these values as a treatment trigger might be as beneficial in predicting hypotension as the HPI performance [38]. This close relationship between the HPI alarm value of 85 and MAP in the range of 70 to 73 mmHg was confirmed in a post hoc secondary analysis of the EU-HYPOROTECT study, which included 702 patients undergoing elective major noncardiac surgery with the use of HPI technology [39]. A study performed on a group of 33 patients showed a high degree of similarity between HPI and MAP [32]. The authors showed a high cross-correlation coefficient of −0.91 ± 0.04 with no time delay between HPI and MAP with negative value due to their inverted trend, meaning that a drop in MAP is accompanied by a rise in HPI. A comparison of the HPI and MAP alarms revealed that the HPI alarms started 3 min earlier before the episode of hypotension with a corresponding MAP value around 71 mmHg. When a threshold of MAP of 70 mmHg or lower was chosen, the HPI alarm signaling upcoming hypotension was present 98% of the time [32]. Another study trying to prove the usefulness of MAP analysis in hypotension prediction compared a change in MAP to a predictor using both absolute MAP values and a change in MAP [40]. This linear extrapolation on current and previous MAP values (LepMAP) analyzed 1, 2 and 5 min before the occurrence of hypotension proved significantly superior to simple MAP change over time. The authors also found a strong predictive ability of single MAP values 1, 2 and 5 min before the episode of IOH in predicting hypotension. The AUC ROC values were significantly higher for the single MAP values than Δ MAP and not statistically different from LepMAP [40].

### Limitations of the Study

Our study has some limitations that should be acknowledged. First, as the retrospective analysis of our study included data derived from the hemodynamic monitor, we do not have information on the quality of the arterial waveform, and both technologies analyzed in the study are so-called arterial pressure-derived calculations. Second, the study also lacks data on whether the anesthesiologist was following the treatment protocols. A complicated hemodynamic protocol, the need to analyze many hemodynamic parameters dynamically changing on the monitor and neglecting the monitor alarms were some of the possible reasons for the failure of the Maheshwari study analyzing the HPI technology, as mentioned before [28]. Finally, due to a small sample size, the data analyzing the postoperative patients’ status does not seem to be informative. However, from the clinical point of view, it is very interesting whether the reduction in hypotension with the HPI technology affects the rate of postoperative organ injury.

In summary, this single-center study showed a significant reduction in the duration and severity of hypotension below 65 mmHg and below 50 mmHg in patients undergoing major abdominal surgery having hemodynamic monitoring with the HPI algorithm in comparison to the FloTrac technology. Due to its retrospective character, the study reflected real-life clinical situations and supported the use of the HPI algorithm in perioperative settings. However, due to the recently published concerns regarding the true HPI usefulness and the bias of data selection, while introducing and developing the HPI algorithm, further randomized clinical trials are needed.

## Figures and Tables

**Table 1:** Basic demographic and epidemiological data.

Characteristics	All Patients, n = 123	FloTrac, n = 61	HPI, n = 62	*p*-Value
Age in years, (mean ± SD)	59 ± 17	59 ± 15	60 ± 18	0.62
Weight (kg) (mean ± SD)	73 ± 20	73 ± 19	71 ± 19	0.75
Height (cm) (mean ± SD)	169 ± 14	169 ± 13	169 ± 16	0.76
Gender male, n (%)	76 (61.7%)	40 (65.6%)	36 (58%)	0.85
ASA classification, n (%)				
I	0 (0%)	0 (0%)	0 (0%)	
II	33 (27%)	16 (26%)	17 (28.5%)	0.67
III	85 (69%)	43 (70.5%)	42 (68%)	0.39
IV	5 (4%)	2 (3.5%)	3 (4.8%)	1.0
Hypertension (n, %)	61 (49.5%)	29 (47%)	32 (52%)	0.72
Coronary artery disease (n, %)	35 (28%)	18 (29.5%)	17 (27%)	0.84
ACE-I (n, %)	48 (39%)	23 (37%)	25 (40%)	0.85
β-blockers (n, %)	41 (33.3%)	20 (33%)	21 (35%)	1.0
Type of abdominal surgery (n, %):				
● Pancreatic tumor	6 (4.8%)	4 (6.5%)	2 (3.2%)	0.43
● Liver tumor	9 (7.3%)	2 (3.2%)	7 (11.2%)	0.18
● Gastrointestinal tumor	64 (52%)	30 (49%)	34 (54%)	0.59
● Inflammatory bowel disease	44 (35.7%)	25 (41%)	19 (31%)	0.26

ASA—American Society of Anesthesiology, ACE-I—angiotensine converting enzyme inhibitors.

**Table 2:** Basic perioperative data.

Characteristics	All Patients, n = 123	FloTrac, n = 61	HPI, n = 62	*p*-Value
Time of hemodynamic monitoring in minutes (mean ± SD)	241 ± 130	220 ± 119	263 ± 119	0.12
Number of patients requiring noradrenaline infusion (n,%)	112 (91%)	56 (92%)	56 (90%)	1.0
Dose of noradrenaline (mcg/kg/min) [median, Q1–Q3]	0.09 [0.05; 0.12]	0.09 [0.06; 0.12]	0.1 [0.05; 0.13]	0.8
Median value of crystalloids during anesthesia (mL) [median, Q1–Q3]	2300 [1900; 2800]	2100 [1700; 2850]	2500 [1800; 2800]	0.48
Blood loss during anesthesia (mL) [median, Q1–Q3]	150 [100; 200]	150 [100; 200]	150 [100; 200]	0.98
Diuresis during anesthesia (mL) [median, Q1–Q3]	500 [300; 800]	500 [300; 800]	500 [300; 800]	1.0
Postoperative CRP values (mg/dL)	7.6 [3.6; 18.4]	9.1 [3.6; 22.5]	6.2 [3.6; 16.6]	0.95
Postoperative creatinine levels (mg/dL)	0.83 [0.7; 1.1]	0.82 [0.66; 1.04]	0.86 [0.74; 1.15]	0.37
Postoperative troponin levels (ng/L)	10 [5; 16]	11 [7; 16]	9.5 [5.7; 13]	0.4
Perioperative mortality (n; %)	13 (10.5%)	7 (11.4%)	6 (9.6%)	0.77

**Table 3:** Data on the hypotensive events for MAP < 65 mmHg in the analyzed groups.

Characteristics	FloTrac Group	HPI Group	*p* Value
Time-weighted average for MAP < 65 mmHg, mmHg (median, Q1–Q3)	0.31 (0.14–0.84)	0.09 (0.01–0.24)	0.000009
Number of patients with hypotensive events < 65 mmHg, n (%)	53 (87%)	46 (74%)	0.11
Number of hypotensive events < 65 mmHg per patient (median, Q1–Q3)	3 (2–7)	2 (0–4)	0.007
Duration of hypotensive events < 65 mmHg in minutes (median, Q1–Q3)	2 (1–6)	2 (1–3)	0.000065
Total time in hypotension < 65 mmHg in minutes (median, Q1–Q3)	12 (4.8–36.1)	4 (0–9.7)	0.000078
Total time in hypotension < 65 mmHg as percentage of the monitoring time (median, Q1–Q3)	6.4 (2.3–16.2)	2.1 (0–4.3)	0.000023

HPI—Hypotension Prediction Index, MAP—mean arterial pressure.

**Table 4:** Data on the hypotensive events for MAP < 50 mmHg in the analyzed groups.

Characteristics	FloTrac Group	HPI Group	*p* Value
Time-weighted average for MAP < 50 mmHg, mmHg (mean ± SD)	0.12 ± 0.31	0 ± 0.01	0.0018
Number of patients with hypotensive events < 50 mmHg, n (%)	16 (26%)	9 (14%)	0.12
Average number of hypotensive events < 50 mmHg per patient (mean ± SD)	1 ± 2	0 ± 0	0.02
Average duration of hypotensive events < 50 mmHg in minutes (mean ± SD)	4 ± 4	2 ± 1	0.19
Total time in hypotension < 50 mmHg in minutes (mean ± SD)	3.3 ± 9.1	0.3 ± 0.8	0.02
Total time in hypotension < 50 mmHg as percentage of the monitoring time (mean ± SD)	2.2 ± 6.8	0.1 ± 0.5	0.02

HPI—Hypotension Prediction Index, MAP—mean arterial pressure.

## Data Availability

The datasets generated and/or analyzed for this study are currently not publicly available due to their in other analyses. Selected data, however, are available from the corresponding author upon request.
